# Rheological, Fresh State, and Strength Characteristics of Alkali-Activated Mortars Incorporating MgO and Carbon Nanoparticles

**DOI:** 10.3390/ma17235931

**Published:** 2024-12-04

**Authors:** Mohammad Ali Hossain, Khandaker M. A. Hossain

**Affiliations:** Department of Civil Engineering, Toronto Metropolitan University, Toronto, ON M5B 2K3, Canada; mohammadali.hossain@torontomu.ca

**Keywords:** alkali-activated mortar, MgO, MWCNT, rGO, rheology, workability, strength, ultrasonic pulse velocity

## Abstract

This study presents a comprehensive assessment of the fresh state, rheological, and mechanical properties of alkali-activated mortars (AAMs) developed by incorporating magnesium oxide (MgO) and nanomaterials. A total of 24 AAM mixes with varying content of MgO, multi-walled carbon nanotube (MWCNT), and reduced graphene oxide (rGO) were developed following the one-part dry mix technique using powder-based activators/reagents. The effects of the types/combinations of source materials (binary or ternary)/reagents, MgO (0 to 5%), MWCNT (0 to 0.6%), and rGO (0 to 0.6%) were evaluated in terms of the mini-slump flow, setting times, viscosity, yield stress, compressive strength, ultrasonic pulse velocity (UPV), and microstructural properties. The results showed that the addition of finer MgO/nano-fillers produced a higher viscosity and yield stress accompanied by a lower slump flow and setting times. The addition of 5% MgO resulted in the lowest slump flow of 80 mm, 2–2.5 times higher viscosity, and the reduction in the initial and final setting times of about 21% and 16%, respectively. Mixes with MWCNT showed about 5–10% higher viscosity whereas for mixes with rGO, the values were noted to be 8% higher, on average, than the mixes with no MWCNT or rGO. All the developed AAMs exhibited shear-thinning behavior. The 28-day compressive strength of the AAMs ranged from 37 MPa to 49 MPa with 5% MgO and up to a 0.3% MWCNT/rGO addition increased the compressive strength. Correlations among the fresh state, rheological, and mechanical properties such as the viscosity, slump flow, setting time, compressive strength, and UPV are also described.

## 1. Introduction

Alkali-activated (AA) binders/geopolymers, which are the product of the reaction of aluminosilicate-rich source materials and alkaline reagents/activators, have proven to be promising new alternatives to concrete binders [1,2,3,4]. Cement-free green geopolymers have attracted considerable attention because of their low permeability, satisfactory initial compressive strength, excellent fire resistance, and good chemical resistance [5,6].

The source materials can be fly ash (FA), ground granulated blast-furnace slag (GGBFS), metakaolin, and other industrial wastes. Alkali activators such as sodium hydroxide (NaOH), potassium hydroxide (KOH), sodium silicate (Na_2_SiO_3_), and potassium silicate (K_2_SiO_3_) are used for the activation of source materials/precursors [7,8,9,10]. Normally, geopolymers are hardened through polymerization instead of hydration as in the case of cement-based binder. The polymerization process involves a rapid chemical reaction in an alkaline solution on Si-Al species, resulting in a three-dimensional polymeric chain-and-ring structure consisting of Si-O-Al-O bonds with the slow growth of crystalline structures [3,4,11,12,13].

AA materials are generally characterized by lower Si and higher calcium content, whereas geopolymers by a low calcium content [3,14,15,16,17,18,19]. Conventionally, AA binders (AABs) are produced by employing a two-part technique using activator solutions and solid precursors while one-part ones use powdered ingredients with water [20,21,22]. Research had been conducted on these materials where the use of FA increased the compressive strength and rate of geopolymerization [19], while GGBFS produced lower slump and higher thixotropic and tensile properties [17,18,19,20,21,22]. Previous research incorporated nanomaterials and self-healing agents to influence the properties of cement-based materials in addition to inducing smart self-healing/self-sensing capabilities [23,24,25,26]. The use of MgO is known to create an autogenous expansion to compensate for the shrinkage and also impart the self-healing of cracks by producing healing products and densification of the concrete microstructure with a high viscosity and increased strengths [24,25,26,27]. AA concrete with a low and high carbon content showed a high and lower shrinkage, respectively [28]. Alkali-activated slag (AAS)–rice husk ash (RHA) paste produced with MgO showed a lower slump flow/workability [29]. MgO-incorporated AA mixes showed a lower setting time with a high viscosity/yield stress and increased compressive strength due to the formation of Mg(OH)_2_ and refined pore structure [30,31].

Concrete structures are exposed to degenerations/deteriorations over time, which need cost-effective monitoring systems. Hence, concrete with self-sensing properties has been developed by using the piezoresistive effect of electrically sensitive carbon nanomaterials such as a carbon nanotube (CNT), graphene, and graphene oxide (GO) dispersed in the matrix forming a conductive network [32,33,34,35,36,37,38]. Multiwall CNTs (MWCNTs) are the most promising reinforcing materials for their exceptional mechanical and self-sensing characteristics to produce the next generation of high-performance nanocomposites if proper dispersion and optimized MWCNT–matrix interfacial interaction are achieved [34,35,36,37].

The addition of a 0.1–0.2% pristine functionalized CNT in a metakaolin-based geopolymer showed a marginal decrease with no significant change in rheology [34]. The use of MWCNT functionalized with a hydroxyl group in a granite-based geopolymer system exhibited a higher yield stress and plastic viscosity associated with a significant fluid loss with time [38]. A decrease in setting times with increased MWCNT content for FA-incorporated geopolymer mixes was noted [39]. A super-high specific surface area of nanomaterials leading to the absorption of a significant amount of free water and reduction in the available binding water was attributed to such changes in the fresh state and rheological properties [38]. Zhong et al. [40] found changes in the rheological properties with GO-incorporated geopolymer composites used in extruded 3-D printing. A notable reduction in the fluidity of slag-based AA mortars (AAMs) with the increase in the GO dosage was observed [41]. The addition of 0.3% and 1% nano graphite decreases the workability due to the thickening effect [42], while the addition of GO up to 0.02% wt. of binder resulted in a reduction in the flowability and setting time for the paste mixes [43].

The rheological behavior of AAMs displayed shear-thinning properties, showing an increase in the yield stress, plastic viscosity, and compressive/flexural strength with the increase in the MWCNT content from 0.3% to 0.35% with minimal compressive strength loss against acid and sulfate attacks with the use of 0.3% MWCNT. This was attributed to the effective bridging action of MWCNT limiting the progression of microcracks in AAM-based nanocomposites provided homogeneous distribution and good MWCNT–paste adhesion are achieved [44,45]

Experimental results showed that the rheological behavior of the fly ash geopolymer paste conformed to the Bingham and Herschel–Bulkley model [46,47]. Taborda-Barraza et al. [48] reported an increase in the yield stress and viscosity in geopolymeric systems incorporating a 0.2 wt.%. CNT content. The addition of CNTs in geopolymer concrete led to improved workability/flowability without compromising the setting time, whereas rGO incorporation affected the workability, suggesting a need for careful dosage optimization to maintain fresh properties [49,50,51]. In contrast, Meng et al. [52] emphasized the importance of judicious mix design and proper dispersion techniques for mitigating segregation in geopolymer concrete containing rGO. Jiang et al. [53] found a reduction in the viscosity and shear-thinning properties in rheological behavior, which was attributed to the lubricating effect of well-dispersed CNTs within the matrix. A similar reduction in the viscosity with rGO incorporation, contributing to improved flow behavior, was observed [54]. Zhu et al. [55] highlighted an increase in the yield stress and plastic viscosity in geopolymer concrete containing CNTs due to the reinforcing effect improving interparticle bonding. Conversely, rGO-incorporated geopolymer concrete exhibited nuanced effects on the yield stress and plastic viscosity, as discussed by Liu et al. [56].

The compressive strength characteristics of AA/geopolymer binders had been investigated and found to be influenced by the type/dosages of the incorporated nanomaterials, precursors, and activators [57,58,59,60]. An increase and a decrease in the compressive strength with the addition of 0.1% and 0.2% of pristine MWCNTs, respectively, was observed for functionalized MWCNTs [34]. The addition of 0.05–0.15 wt.% functionalized MWCNTs and replacement of 10–40 wt.% of FA with GGBFS showed an increase in the compressive strength of geopolymer pastes, with 0.1% MWCNTs and 30% GGBFS showing the highest increase [57]. The compressive strength of FA-based geopolymers steadily increased with the increase in the graphene nanoplatelets (GNPs) content from 0.1 to 1 wt.% [58], while a steady decrease with the increase in GO content from 0 to 0.12% was observed for 6 wt.% NaOH-activated slag [59]. Matalkah and Soroushian [60] reported a decrease in the compressive strength of AABs based on a ternary blend of FA, GGBFS, and albite using 0.1 and 0.3 vol% of GNPs while a little increase with 0.2 vol%.

The previous research on the rheological properties of AA/geopolymer mixes incorporating CNT/MWCNT suggests no significant studies using rGO or MgO. Most of these studies lack a detailed characterization of the fresh state properties of the AA/geopolymer system containing nanomaterials (such as MWCNT or GO) and self-healing agents like MgO. Moreover, the prevalent standards do not provide any specific directives to assess the change in the complicated fresh state properties of conventional geopolymer systems after adding nanoparticles [38]. Most former studies on the effect of the healing agent or nanomaterials addition on AA/geopolymer mix systems have focused on assessing the mechanical, microstructural, durability (crack-healing), and conductive (self-sensing) performances [34,35,36,37,38]. In addition, most of the currently available literature mostly presents the rheological performance of AA/geopolymer mortars prepared using a two-part (wet mix) rather than one-part (dry mix) technique.

This paper focuses on the development and evaluation of the rheological, fresh state, and mechanical properties of AA mortars (AAMs) containing healing agents (such as MgO) and nanomaterials (MWCNT and rGO). The novel aspects of the study addressing the research gaps are as follows: the use of binary and ternary combinations of FA and GGBFS as precursors, two combinations of reagents (type 1 and type 2), high-calcium-based activators/reagents rather than traditional Na-based ones, and one-part dry mix technique as well as a comparative performance study considering variable mix design parameters and materials. The influences of the mix ingredients, such as the combinations of reagents, binary/ternary blends with fundamental chemical ratios present in the precursors/reagents, and MWCNT/rGO/MgO dosages on the fresh state (mini-slump flow/variation with time and setting times), rheological characteristics (viscosity/variation time, yield stress, shear-thinning, and rheopectic behavior), and mechanical properties (compressive strength and ultrasonic pulse velocity ‘UPV’) have been described with reference to microstructural aspects. Moreover, correlations among the fresh state, rheological, and mechanical properties (viscosity–slump flow, slump flow–setting time, viscosity–compressive strength, compressive strength–UPV) are developed. The results of this study will benefit engineers and scientists to understand more about the mix design and properties of developed AAMs to further develop and characterize mixes for better performance and utilize them as base materials to develop AA concretes (incorporating coarse aggregates) and fiber-reinforced composites with self-healing and self-sensing abilities (with MgO, MWCNT, and rGO).

## 2. Experimental Program

A total of 24 AA mortar (AAM) mixes were developed and assessed for the fresh state, rheological, and mechanical properties. AAM mixes were prepared as per the “one part-dry mix” method using two combinations of powder-based reagents as activators. Three different filler/additive materials, namely MgO, MWCNT, and rGO, were included to observe their individual influence on the AAM properties.

### 2.1. Materials

The ambient-cured AAM mixes were prepared using two combinations of FA and GGBFS as source materials. The binary (designated as B) AAM mixes were prepared by mixing high-calcium FA type C (FA-C) and GGBFS, whereas the ternary ones (designated as T) were developed by mixing high-calcium FA type C (FA-C), low-calcium FA type F (FA-F), and GGBFS. The rationale for selecting these industrial waste-based source materials is to reduce CO_2_ emissions and improve the AAM properties (particularly strength) from their high-calcium and a considerable amount of silicate contents. A high-range water reducer admixture (HRWRA) was required to ensure flowable AAM mixes. A polycarboxylate ether-based superplasticizer with a solid content of 40%, pH of 6, and specific gravity of 1.06 g/cm^3^ was used. Silica sand with a maximum particle size of 600 μ was used as fine aggregate. The MgO used in this study was prepared by burning MgCO_3_ for two hours at 900 °C, classified as lightly burnt [24]. AAM mixes were prepared by using MWCNT and rGO nanomaterials at different dosages. MWCNTs had a diameter of 20–30 nm, length of 10–30 μm, Blaine fineness of 110 m^2^/g, density of 1.2 g/cm^3,^ and electrical conductivity of greater than 10^−2^ s/cm with 95% or more purity. rGO (black powder form with a purity of 99% or more) with 80–10 layers, a thickness of 3–6 μm, a diameter of 0.5 µm, density of 0.03 g/cm^3,^ and Blaine fineness of 130 m^2^/g was used. The physical properties and chemical compositions of FAs, GGBFS, silica sand, and MgO are presented in Table 1.

As per the dry mix technique, two combinations of different alkaline reagents were used in powder form (obtained from Westlab, Surrey, BC, Canada) to prepare two types of high-calcium-based activators to activate the source materials. Activator type 1 was prepared by mixing calcium hydroxide (CaOH_2_ with specific gravity = 2.24 and pH = 12.4–12.6) with sodium meta silicate (Na_2_SiO_3_.5H_2_O with modular ratio, SiO_2_/Na_2_O = 1, specific gravity = 0.7, pH = 11.3) in a ratio of 1:2.5 (CaOH_2_:Na_2_SiO_3_.5H_2_O = 1:2.5) and the modular ratio (SiO_2_/Na_2_O) of this mixture was 3.22. Activator type 2 was prepared by combining calcium hydroxide (CaOH_2_) and sodium sulfate (Na_2_SO_4_ with specific gravity = 2.70 and pH = 7) beads/powder in a ratio of 2.5:1 (CaOH_2_:Na_2_SO_4_). The physical and chemical properties of both activator combinations are listed in Table 2.

### 2.2. AAM Mix Proportions

This extensive experimental study consisted of a total of 24 AAM mixes with two types of muti-component activators (Type 1 and Type 2) and three different fillers/additive materials (MgO, MWCNT, and rGO). All AAM mixes prepared following the “one part” or “dry mix” technique are shown in Table 2. Four control mixes without MgO, MWCNT, and rGO additions were produced. A total of 20 AAM mixes containing MgO, MWCNT, and rGO were developed and their fresh state and rheological properties were compared with those of the four control AAMs developed in a previous study [4,23,45,46,47]. The binary (B) mixes contained the combinations of FA-C with GGBFS, whereas the ternary (T) mixes were prepared with FA-C, FA-F, and GGBFS. The FA contents varied from 52% to 60%, GGBFS varied within 38% to 45%, and silica sand content of 30% by mass of the total binder content (Table 2). The water to binder ratio for the mixes ranged between 0.35 and 0.4. A constant dosage of HRWRA (0.02% wt of binder) was used to induce the required workability and to prevent the interference of its acidic nature on changing the proportions of alkaline reagents.

The AAM mixes incorporating MgO were prepared using 5% MgO by weight of the total binder content. The mixes with CNT/rGO were prepared by incorporating 0.3 and 0.6% MWCNT/rGO by wt. of binder contents. All the activator components and the initial chemical ratios in the mix compositions are presented in Table 3. Activator type 1 has a reagent component ratio (calcium hydroxide to sodium metasilicate) of 1:2.5, while activator type 2 has a reagent component ratio (calcium hydroxide to sodium sulfate) of 2.5:1. These ratios were chosen based on previous research studies on AABs [4,22,61,62,63]. The fundamental chemical ratios in terms of silicon oxide to aluminum oxide, sodium oxide to silicon oxide, calcium oxide to silicon oxide, and sodium oxide to aluminum oxide were evaluated from the chemical compositions of the reagents and source materials. These chemical ratios were found to be within the range of AAMs as per recent studies on fly ash and slag-based mortars [4,22,61,62,63].

### 2.3. Mixing Sequence

The powdered reagent components were first mixed thoroughly and then added to the rigorously blended source materials. The complete binder system was then dry mixed for about 5 min in a shear mixer before two-thirds of the required water was gradually added to the mix while mixing continued for 3–4 min. Then, HRWRA mixed with the remaining amount of water was gradually added for 4–5 min to make a flowable paste. After that, silica sand was added slowly for 3–4 min as per the proportions given in Table 2 and mixed for an additional one minute to make control AAMs.

**Figure 1:**
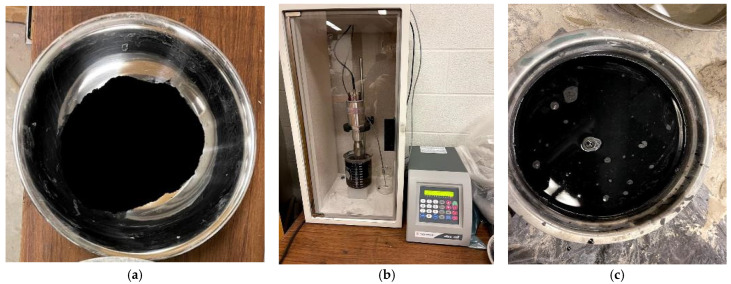
MWCNT (**a**) before sonication, (**b**) during sonication, and (**c**) after sonication.

For AAM mixes with the MWCNT and rGO, two-thirds of water and 50% HRWRA were mixed for 1–2 min by hand stirring in the beaker, then MWCNT/rGO was added and hand stirred for 2–3 min. The beaker was then placed inside the sonicator, the probe was inserted in the beaker, and sonication was performed for 30–40 min. The amount of sonication energy was 50 J/mL~75 J/mL to disperse MWCNT/rGO to the water [12,13]. The mixture of sonicated MWCNT/rGO with water and HRWRA was then slowly added into the already made dry mix (as explained previously in control mix preparation) for 4–5 min while mixing continued in the shear mixer followed by the gradual addition of silica sand to prepare MWCNT/RGO-incorporated AAMs. Figure 1 shows the MWCNT before, during, and after sonication and the sonication device. Testing of the fresh state properties was performed immediately after the AAMs were made.

### 2.4. Test Methods and Testing Procedures

**Figure 2:**
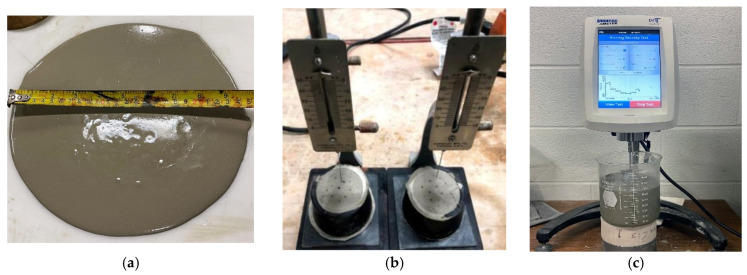
Measurement of (**a**) slump flow using mini-slump cone, (**b**) setting times using V-cat apparatus, and (**c**) rheology using Brookfield DV3T rheometer.

The slump flow measurements of AAMs were made using a mini-slump cone (Figure 2a) at 0, 30, and 60 min post-mixing as per ASTM C1437 [64]. The initial and final setting times of the mixes were determined as per ASTM C 191-a [65] using V-cat apparatus, as shown in Figure 2b.

The rheological characteristics of the developed AAM mixes were determined based on the yield stress and plastic viscosity along with their thixotropic properties. A DV3T rheometer from Brookfield (Middleborough, MA, USA) was used for viscosity measurements using an RV-04 spindle for 10 min to obtain a stable viscosity value once the torque became constant (Figure 2c). The yield stress test was also conducted using the vane spindle V-73. Both the yield and viscosity tests were conducted at the same rotational speed of 3.5 RPM.

**Figure 3:**
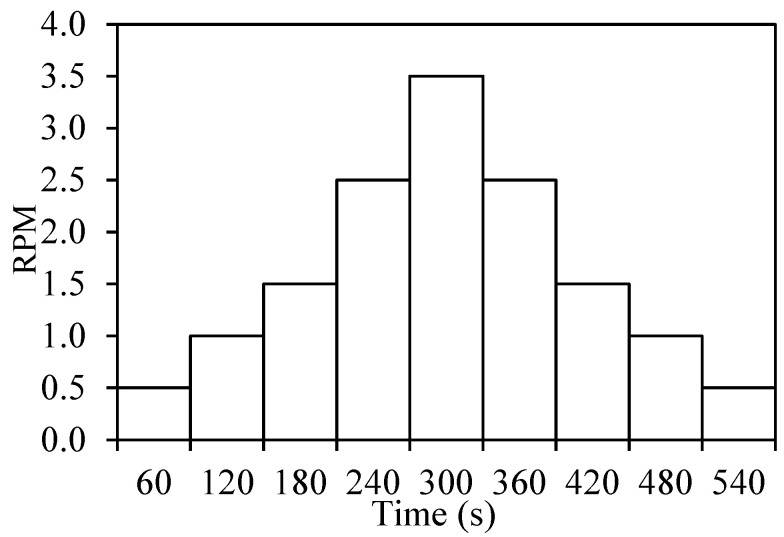
Rheology test program for hysteresis loop behavior.

A rheological procedure was defined separately to observe the hysteresis loop and thixotropic behavior for the viscosity of the AAM mixes. The program for the hysteresis loop behavior consisted of two phases for the shear rate in revolutions per minute (RPM)—ramp up and ramp down, as indicated in Figure 3. The RPM was gradually increased from 0.5 RPM to 3.5 RPM and then steadily decreased from 3.5 RPM to 0.5 RPM in the ramp down stage. Each RPM was administered for a period of 60 s using the vane spindle V-73. The thixotropic behavior was assessed by a stepped viscosity program using a V-73 spindle in which viscosity measurements were made for 5 min duration at 0, 30, and 60 min post-mixing with the spindle rotational speed of 3.5 RPM.

The compressive strength was determined at 28 days by testing 50 mm × 50 mm × 50 mm cube specimens as per ASTM C109 [66]. An ultrasonic pulse velocity (UPV) test was conducted on the same cube specimens using a Portable Ultrasonic Non-Destructive Digital Indicating Tester as per ASTM C597 [67].

## 3. Results and Discussions

### 3.1. Effect of MgO, MWCNT, and rGO on Plastic Viscosity and Yield Stress

#### 3.1.1. AAM Mixes with 5% MgO

**Figure 4:**
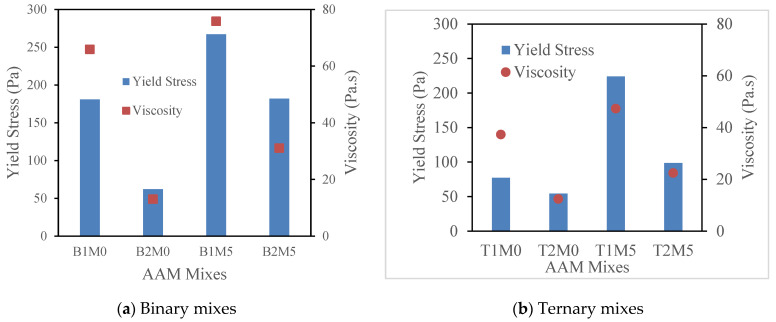
Variations in plastic viscosity and yield stress for AAM mixes with 5% MgO.

Figure 4 portrays the variations in the yield stress and plastic viscosity of the binary and ternary AAM mixes with 5% MgO. In general, all the AAM mixes (control and with MgO alike) with reagent type 1 exhibited higher viscosity as compared to their counterparts with reagent type 2. The mixes with reagent type 1 (both binary and ternary) showed about a 3.0 to 3.5 times higher plastic viscosity compared to those with type 2 reagent. Mixes with reagent 1 contained higher SiO_2_/Al_2_O_3_ compared to those with reagent type 2, as shown in Table 3, which can be attributed to the higher viscosity of AAMs (B1M0, B1M5, T1M0, and T1M5) with reagent 1 (Figure 4). As observed by Palacios et al. [68] with solution-based reagents, high SiO_2_/Al_2_O_3_ leads to higher colloidal Si-O-H-Na formation and thus contributes to denser mixes. Now, comparing the binary and ternary mix compositions, the presence of fly ash type F in the ternary AAM mixes as the source material was observed to contribute to the high viscosity values. This is because of the round-shape characteristics of fly ash F particles and their fewer in-particle frictions under shear force.

Moreover, it was reported and reasoned that an increase in the silicate content as compared to the alkaline hydroxide contributes towards high viscosity and high yield stress values up to a certain point. This is when the rheological behavior of an FA-based geopolymer/AA system changes from Newtonian to a non-Newtonian one [4,22]. This factor can arguably affect the AAM mixes with reagent type 1, in general, (which contain higher silica, as shown in Table 2 and Table 3) to give a higher viscosity. As the binary mixes and AAM mixes with reagent type 1 appear to be more viscous, a higher shear stress will be required to break the particles and reach the failure limit. Thus, it can be referred to as the reason for the high yield stress values for the binary AAM mixes and AAM mixes with reagent type 1. Introduction of the FA-F as the source material for the ternary mixes can be considered for situations with improved workability.

Now, considering the binary mixes as shown in Figure 4a, the control AAM mixes (B1M0 and B2M0) showed about 14% and 58% lower viscosity values as compared to those prepared with 5% MgO agent for reagent type 1 and type 2, respectively. Similarly, for the ternary mixes as presented in Figure 4b, the reduction in the viscosity of the control mixes was shown to be about 21~22% and 40% compared to the AAM-MgO mixes with reagent type 1 and type 2, respectively. The high viscosity of the AAM mixes with MgO can be attributed to a higher solid portion resulting from the MgO addition. The MgO with a fine surface area absorbs more water, resulting in more viscous mixes [27]. This finding also matches with the observed trend of the fresh state properties of the AA system assessed by Hwang et al. [69], where a decrease in flowability was noted with an increase in the MgO content. This can also be explained by the rapid reaction of MgO, forming the hydrotalcite-like phase (Mg_6_Al_2_(OH)_16_CO_3_.4H_2_O) and C-S-H gel, for a denser matrix, early strength levels, and faster initial setting [70,71]. Moreover, the finer MgO particles react with more water to form worm-like Mg(OH)_2,_ resulting in refined pores and higher viscosity [31].

Similarly, in the case of the yield stress values, the binary mixes with 5% MgO exhibited about 2.0–2.5 times higher yield stress values, on average, when compared to their control counterpart with 0% MgO (Figure 4). However, the ternary AAM mixes with 5% MgO showed about 2.5 times higher yield stress values in comparison with the mixes prepared with 0% MgO (T1M0 and T2M0). These high yield stress values for MgO-added AAM mixes can be attributed to more water absorption by MgO, resulting in relatively viscous mixes and, therefore, a higher shear stress value to reach the yielding point. The comparatively lower yield values for the ternary mixes were due to quicker shear deformation at lower stress values resulting from the presence of round-shaped fly ash F within the source material.

#### 3.1.2. AAM Mixes with 0.3% and 0.6% of MWCNT

**Figure 5:**
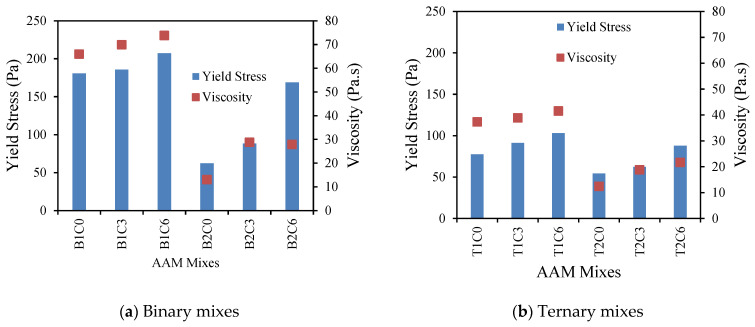
Variation in viscosity and yield stress for AAMs with MWCNT.

Figure 5a,b show the effect of the MWCNT content on the plastic viscosity and yield stress of the AAM mixes (binary and ternary). As can be illustrated, all the mixes prepared using reagent type 1 showed relatively higher viscosity and yield stress values as compared to those prepared with reagent type 2 due to the formation of more colloidal Si-O-H-Na. Moreover, almost all the ternary mixes with both 0.3% and 0.6% MWCNT showed lower viscosity in nature and, thus, relatively lower shear stress values because of the usage of fly ash type F as a source material. This characteristic is more pronounced for binary and ternary AAM mixes with reagent type 1 than AAM mixes with reagent type 2.

The addition of MWCNT resulted in higher viscosity and, thus, high yield stress values as observed in previous studies by Nazar et al. [44]. In the case of the binary mixes (Figure 5a), the addition of 0.3% MWCNT resulted in about a 5.5% and 55% increase in the viscosity values for AAMs with reagent 1 and 2, respectively, as compared to the control AAM mixes with 0% MWCNT. However, the increase in the viscosity was about 5–6% for an increase in the MWCNT content from 0.3% to 0.6% wt. of binder. As for the ternary mixes (Figure 4b) with reagent type 1, the increases in the viscosity values were about 5% and 12% for a change in the MWCNT content from 0% to 0.3% and to 0.6%, respectively. As for reagent type 2, the increments were 58% and 77.5% for the AAM mixes with 0.3% and 0.6% MWCNT, respectively, as compared to the control ones with 0% MWCNT. This can be attributed to the increase in the amorphous phase as pointed out by Khater and Gawaad [36]. As per Li et al. [39] and Parveen et al. [72], the high surface area of the functionalized MWCNT often absorbs quite a significant amount of water, stimulating the geopolymerization/alkali-activation process, and also reduces the workability of the mixes. The liquid content and the solid to liquid ratio also have a significant effect on the viscosity. Now, with the increasing MWCNT content, more water will be absorbed, resulting in more inter-particle friction. This, eventually, results in a reduction in the flowability and, thus, increase in the viscosity. This finding further proves the point that MWCNTs, present in a significantly high amount within a mix, will impact the viscosity, as similarly observed by Macleod et al. [73] in the case of concrete with a CNT liquid admixture. Moreover, the variation in the viscosity with the increasing MWCNT content for the AAM mixes with reagent type 1 was observed to be less pronounced when compared with their counterparts with reagent type 2 (Figure 5a,b). This can be attributed to the high viscous nature of AAM mixes with reagent type 1 resulting from colloidal Si-O-H-Na formation. Now, due to the highly viscous characteristics of mixes with MWCNTs, high shear stress values will be required to reach the yield limit in comparison with the control mixes, as evident in Figure 5a,b.

#### 3.1.3. AAM Mixes with 0.3% and 0.6% rGO

**Figure 6:**
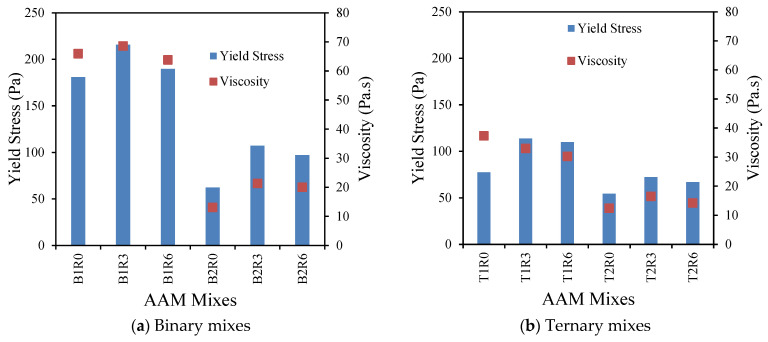
Variation in plastic viscosity and yield stress for AAM mixes with rGO.

The effect of rGO on the variation in the plastic viscosity and yield stress values of AAMs is shown in Figure 6a,b. Like the AAM-MWCNT mixes, mixes with rGO were prepared with varying rGO content of 0.3% and 0.6% wt of binder. From Figure 6a, it is evident that, for reagent type 1, an increase in the rGO content from 0.3% to 0.6% resulted in about a 5.5% and 12% decrease in the viscosity and yield stress values, respectively, as observed by Yang et al. [54]. As for the mixes with reagent type 2, the reductions in the viscosity and yield stress values were shown to be 6% and 9%, respectively. Although the increase is not a significant amount or conclusive, this trend can be attributed to the agglomeration nature of rGO. As described by Liu et al. [74] and Wang et al. [75], rGO has a high tendency for agglomeration, which results in increased particle concentration. Therefore, the viscosity of the mix is increased, and exhibits shear-thinning behavior. However, with respect to the control mixes with 0% rGO, both the viscosity and yield stress values were observed to be increased for AAMs with 0.3% and 0.6% of rGO. Now, a similar trend of variation in the viscosity and yield stress values was noted for all the binary and ternary mixes, as displayed in Figure 6a,b, with the binary mixes showing a higher viscosity and yield stress compared to their ternary counterparts.

#### 3.1.4. Correlation Between Plastic Viscosity and Yield Stress of AAMs

**Figure 7:**
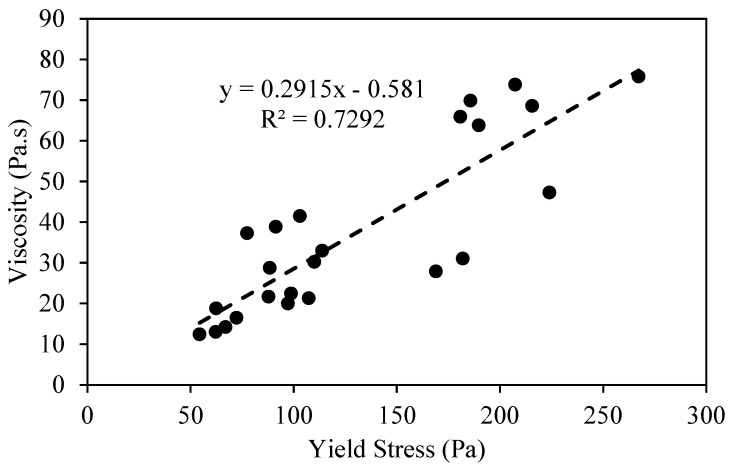
Correlation between plastic viscosity and yield stress of AAM mixes.

Figure 7 shows a linear correlation between the plastic viscosity and the yield stress values of the AAM mixes. The viscosity increases with the increase in the yield stress as per Equation (1):

Viscosity (Pa·s) = 0.2915 (Yield Stress) − 0.581 (1)

A correlation coefficient (R^2^) value of 0.7292 represents a good correlation between the viscosity and yield stress of AAM mixes incorporated with MgO, MWCNT, and rGO.

### 3.2. Initial and Final Setting Times of AAM Mixes

**Figure 8:**
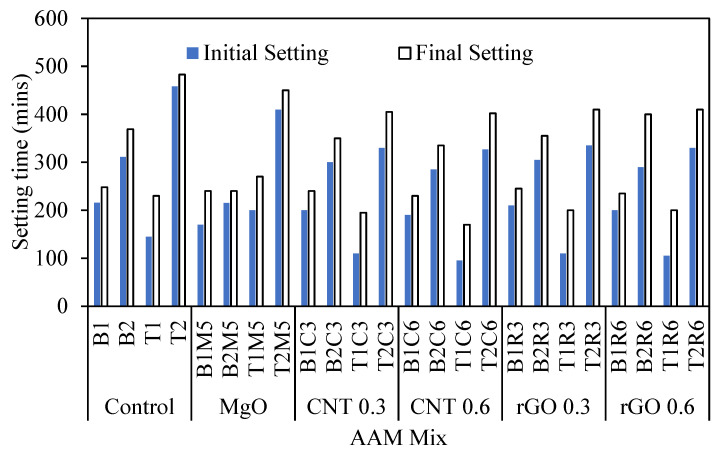
Relationship between initial and final setting time of AA mixes.

Table 3 summarizes and Figure 8 displays the effect of MgO, MWCNT, and rGO on the initial and final setting times of the AAM mixes. It is evident that the addition of MgO and MWCNT yields comparatively lower initial and final setting times compared to their control counterparts. The initial setting of the reagent 1 mixes decreased from 216 min to 190 min, while for the reagent 2 mixes, it decreased from 311 min to 285 min as the MWCNT dosages increased from 0% to 0.6%. The final setting of the reagent 1 mixes decreased from 248 min to 230 min, while for the reagent 2 mixes, it decreased from 369 min to 335 min as the MWCNT dosages increased from 0% to 0.6% (Table 3). Both the MgO and functionalized MWCNT have high water affinity due to an increased surface area. Therefore, a significant quantity of water becomes absorbed within the surface of this material, resulting in a more viscous matrix (as can be seen from the lower mini-slump values with the addition of MgO and MWNCT in Table 3) and, thus, producing lower setting times. However, for mixes with rGO, the variations in the setting times were insignificant as compared to the control mixes, owing to the probable cause of less water affinity of rGO particles ensuring higher workability with low viscosity causing a lower variation in the setting times. However, accelerated hydration of the geopolymer binder by nanoparticles [74,75] can be considered as a reason for the somewhat lesser workability of AAMs with rGO as compared to the control ones.

Moreover, the AAM mixes with reagent type 1 show comparatively lower setting times, which can be attributed to the higher viscosity of those mixes due to the presence of high SiO_2_/Al_2_O_3_ content (Figure 8), which can facilitate quicker dissolution and gel product formations [16]. Hadi et al. [76] observed similar trends for mixes with a varying fly ash content and silicate ratio.

### 3.3. Evaluation of Shear-Thinning Behavior of AAMs Using Hysteresis Viscosity Loop

**Figure 9:**
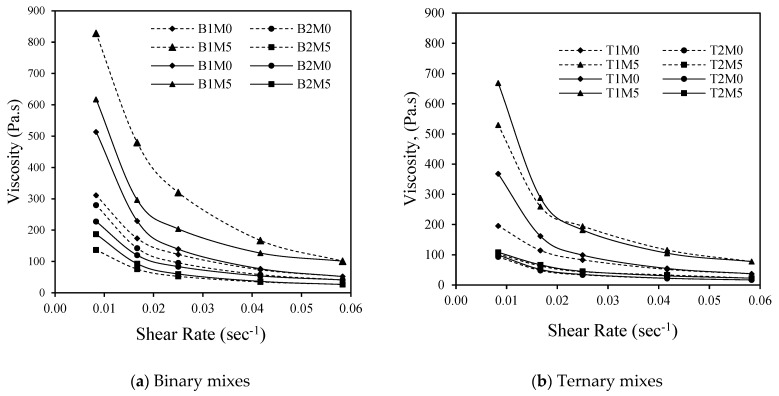
Variation in plastic viscosity and yield stress for AAM mixes with 5% MgO.

**Figure 10:**
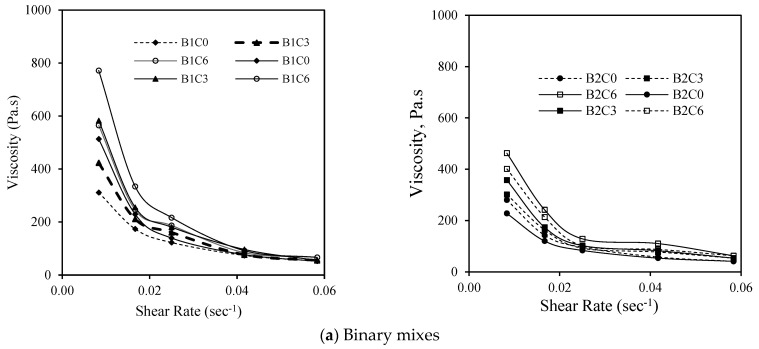

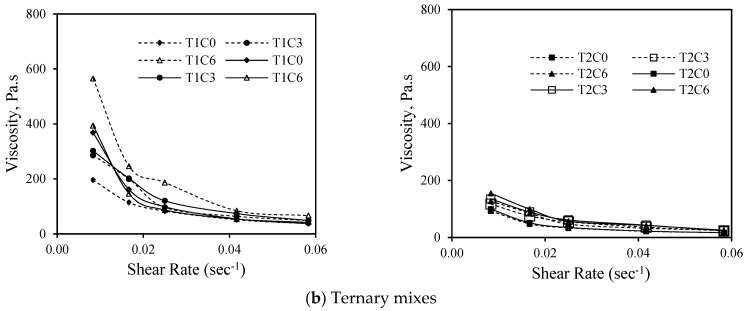
Variation in plastic viscosity and yield stress for AAM mixes with MWCNT.

**Figure 11:**
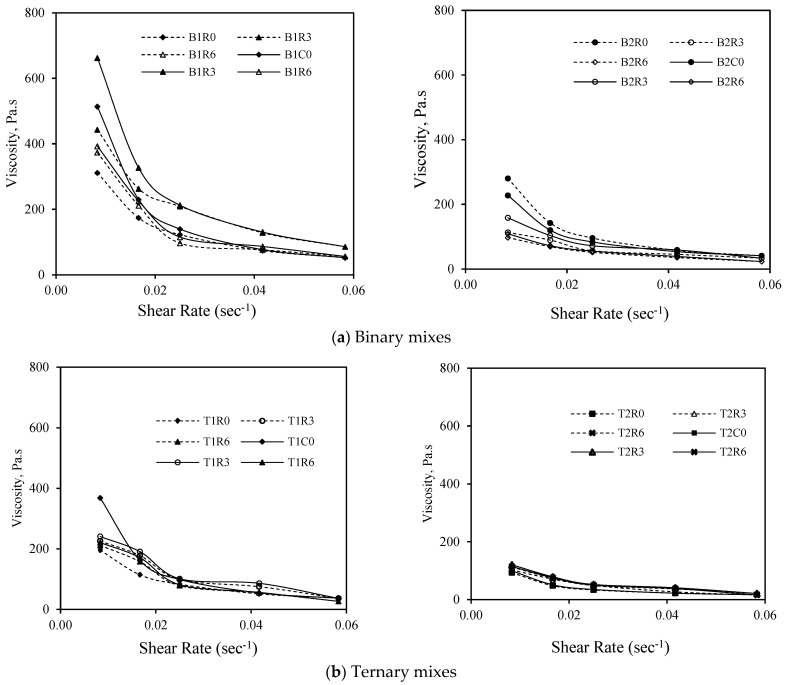
Variation in plastic viscosity and yield stress for AAM mixes with rGO.

Figure 9, Figure 10 and Figure 11 depict the performance of the AAM mixes when subjected to a hysteresis test by increasing the shear rate up to 0.6 and then reducing the shear rate to 0.1. The purpose of this test was to evaluate the shear-thickening or shear-thinning characteristics of the developed AAM mixes. The reduction in viscosity with an increase in the shear rate (0.5 to 3.5 RPM or 0.01/s to 0.06/s) represented by a solid line (shear ramp up) and the gain in viscosity with the decrease in the shear rate (3.5 to 0.5 RPM or 0.06/s to 0.01/s) indicated by the dotted line (shear ramp down) are presented in Figure 9, Figure 10 and Figure 11.

The AAM mixes with 5% MgO showed a decrease in the viscosity with the increase in the shear rate from 0.01 to 0.06 and an increase in the viscosity with the decrease in the shear rate from 0.06 to 0.01, reaching almost equal or somewhat higher values (Figure 9). This characteristic can be depicted as shear-thinning or pseudoplastic behavior. The increase in the viscosity can be associated with the increase in the solids loading (low w/b ratio) in the activated system [38,39,68]. This signifies that at a higher shear rate, the degree of re-flocculation of particles is not significant enough to affect the viscosity of the mixes as opposed to typical cement-based ones. At higher shear rates, cement suspensions become shear thickening (increase in apparent viscosity at high shear rates), often observed in the presence of superplasticizers. In the case of a particulate medium, the particles formed during at-rest conditions are broken down with the increasing shear rate/strain, which, however, retains its form by re-flocculation after a critical strain value [75,77]. In the case of a geopolymer/AA system under increasing shear, flocs with short-range interaction forces among FA particles are formed from the disrupted structure of the long-range order of FA particles. In addition, the shearing stress produced from the hydrodynamic motion of the viscous-activating media affects the shear-thinning characteristics [77].

As for the mixes with the MWCNT (Figure 10) and rGO (Figure 11), a similar trend of change in the viscosity was observed with the increasing shear rate identifying these mixes as well as shear-thinning ones. Despite having a high agglomeration tendency for the MWCNT and rGO, under a high shear rate, the re-agglomeration of these particles will have less to no effect on the viscosity of the developed geopolymer/AA mix systems. Moreover, trends of higher viscosity values were observed for the AAM mixes with reagent type 1, 5% MgO, and MWCNT.

In general, a more rapid decrease in the viscosity was observed for mixes with reagent 1 till 0.02/s (1 RPM), probably due to the higher dissolution potential of sodium metasilicate, leading to the faster release of water during the initial periods of alkali activation. All mixes showed decreased viscosity with an increase in the shear rate, showing pseudoplastic behavior. A similar trend was noted in a previous study where the two-part fly ash-based binders exhibited similar flow curves in both directions (shear ramp up and ramp down) [76]. This reflects that these AAMs are not influenced by the de-clustering of flocci, which is a typical phenomenon in cement-based materials [76].

### 3.4. Evaluation of Thixotropic/Rheopectic Behavior: Viscosity Variation with Time

**Figure 12:**
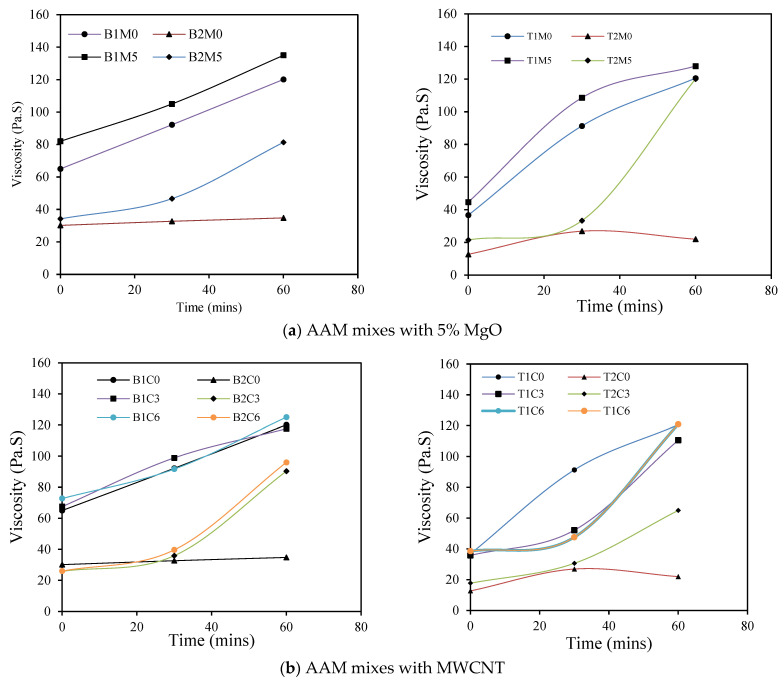

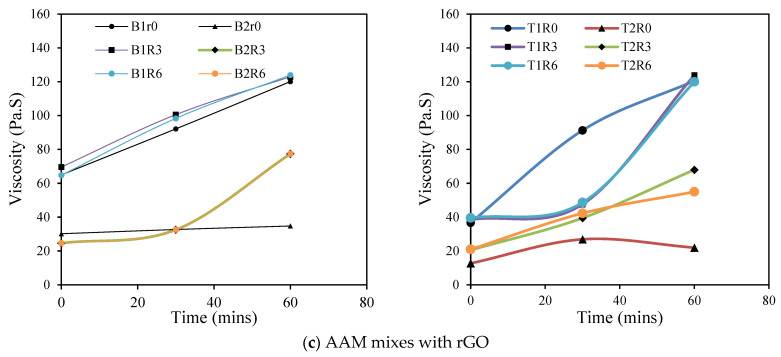
Variation in viscosity with time for different AAM mixes.

The thixotropic behavior of the developed AAM mixes was assessed by subjecting them to a constant shear rate for 0 min, 30 min, and 60 min. Figure 12a–c are a graphical representation of the viscosity evolution over time. In the case of all the AAM mixes prepared with MgO, the viscosity values increased with an increase in the time. For mixes with reagent type 1, the increase was more prominent but at a steady rate, whereas those with reagent type 2, the increase was sharp from 30 min to 60 min but exhibiting lower viscosity values than ones with reagent type 1. This behavior can be classified as “rheopectic” behavior where the viscosity increases with the time. However, for B2M0 and T2M0, the viscosity was observed to be almost the same or somewhat less for an increase in time from 30 min to 60 min. As for the mixes with the MWCNT and rGO, a similar trend of an increase in the viscosity was observed as the time increased from 0 min to 30 min and then 30 min to 60 min, depicting similar “rheopectic” behavior.

### 3.5. Evaluation of Workability of AAMs: Mini-Slump Flow Variation with Time

**Figure 13:**
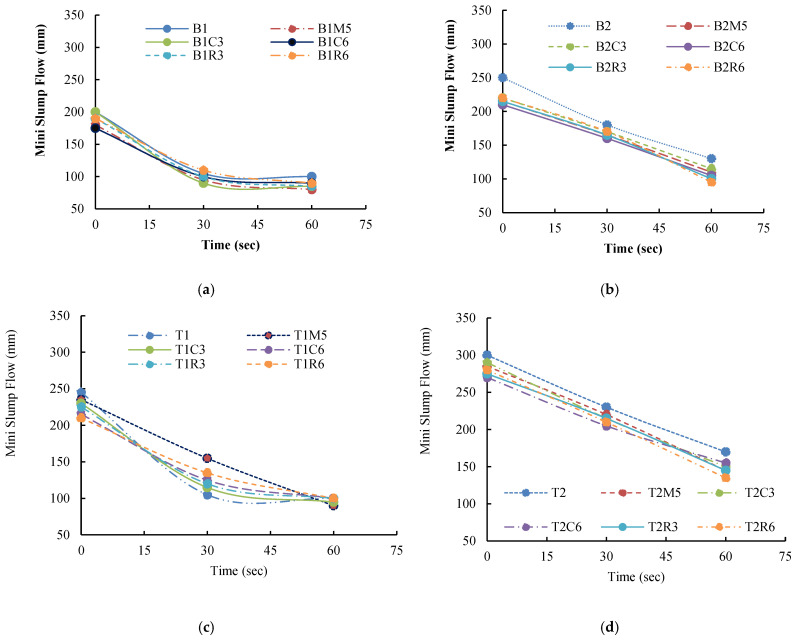
Mini-slump flow vs. time of AAMs (**a**) B1, (**b**) B2, (**c**) T1, and (**d**) T2.

Figure 13 shows the variation in the measured mini-slump flow with time (0 min, 30 min, and 60 min after mixing). All the mixes showed a similar trend of a decrease in the mini-slump values with the increase in the elapsed time, as expected. The ternary mixes (T) exhibited a higher slump flow than their binary counterparts (B) due to the presence of FA-F with round particles. Also, mixes (both binary and ternary) with reagent 2 (B2 or T2) showed a higher slump flow than their reagent 1 counterparts (B1 or T1) for reasons discussed in connection with the viscosity. The mini-slump flow generally decreased with an increase in the MWCNT content whereas the MWCNT mixes showed less slump flow compared to their rGO-incorporated counterparts. In general, the addition of MgO and MWCNT in the control AAMs resulted in a decrease in the slump flow. All the AAMs exhibited a satisfactory initial slump flow (at 0 min), ranging between 175 mm and 300 mm (Table 3 and Figure 13), and showed their potential to retain and regain their workability with time as observed from their shear-thinning or pseudoplastic behavior. However, more investigations are needed to optimize the dosages and combinations of reagent types and nanomaterials.

**Figure 14:**
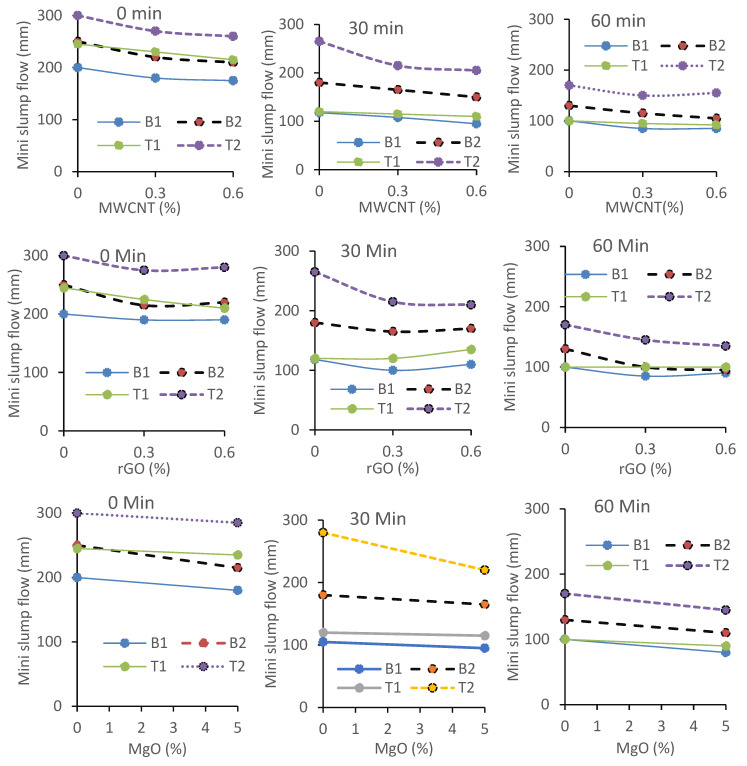
Influence of MWCNT, rGO, and MgO on mini-slump flow of AAMs.

The production of C-A-S-H and C-S-H with shorter Si-Al linkages in both the binary/ternary mixes with reagent 2 (Figure 13 and Figure 14) produced a higher slump flow with a lower viscosity and yield stress (as confirmed from the rheology test results presented earlier) compared to their reagent 1 counterparts, where the formation of N-C-A-S-H and N-A-S-H gels with comparatively longer polymeric chains in addition to C-A-S-H were found to be dominant, as confirmed in research by Sood and Hossain [61].

### 3.6. Relations Among Mini-Slump Flow, Viscosity, and Setting Time

**Figure 15:**
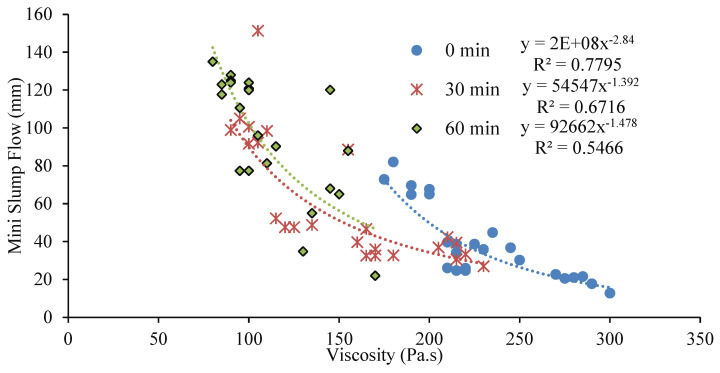
Relation between slump flow and viscosity at 0 min, 30 min, and 60 min.

**Figure 16:**
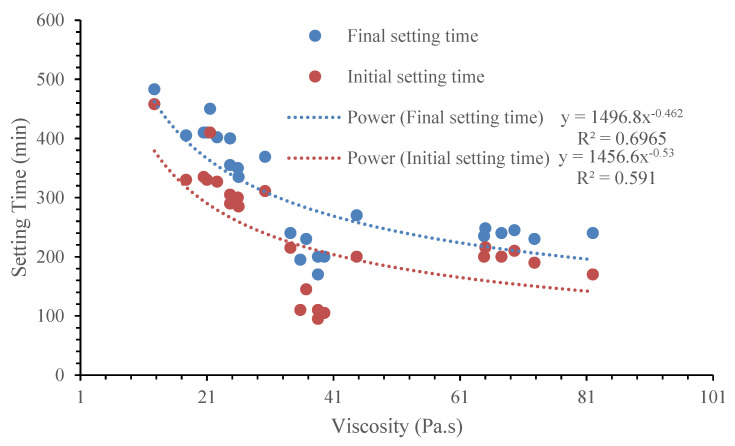
Relation between setting time and viscosity of AAMs.

Figure 15 shows the relation showing the general trend between the measured mini-slump flow and viscosity values at 0 min, 30 min, and 60 min after mixing. The non-linear relationship indicates that a mix with higher viscosity will yield a lower slump flow diameter. This is because of the presence of high in-particle friction/bond resulting from the particle shape, water absorption, and surface area in highly viscous mixes. This particular trend of a decrease in the slump flow with an increase in the viscosity can be observed in Figure 14 where there are correlation coefficient (R^2^) values of 0.7795, 0.6716, and 0.5466, respectively, for 0 min, 30 min, and 60 min. However, the correlation coefficient seemed to decrease with the increase in the elapsed time. Figure 16 also shows a non-linear relationship, indicating a decrease in both setting times (initial or final) with an increase in the viscosity of AAMs incorporating MgO, MWCNT, and rGO.

### 3.7. Compressive Strength and Ultrasonic Pulse Velocity (UPV) of AAMs

Table 3 summarizes the 28-day compressive strength and UPV values of the AAMs. The 28-day compressive strength ranges from 37 MPa to 49 MPa, while the UPV values range between 3354 m/s and 3995 m/s.

**Figure 17:**
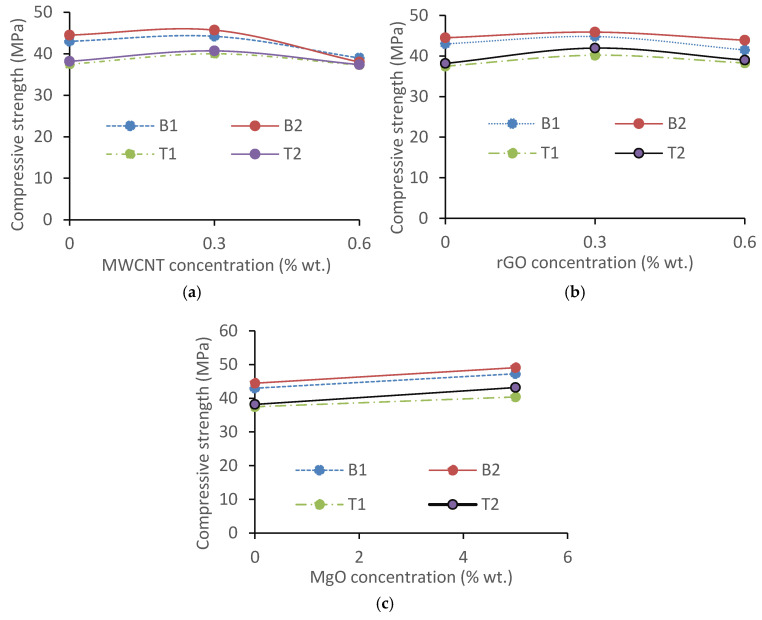
Influence of MWCNT, rGO, and MgO on the 28-day compressive strength of AAMs: (**a**) MWCNT, (**b**) rGO, and (**c**) MgO.

Figure 17a,b show the compressive strength enhancement in the AAMs up to 0.3% MWCNT/rGO and a decreasing trend at a higher dosage, indicating 0.3% is an optimum. A 0.3% MWCNT addition increased the compressive strength by 2.7% (for B2C3) and 6.67% (for T1C3), while for 0.6% MWCNT addition, there was a decrease of 0.53% (for T1C3) and 14.1% (for B2C3). The use of smaller-sized MWCNTs was found to result in a higher mortar compressive strength due to the much finer scale distribution of the MWCNT and filling of the nanopore space within the matrix more efficiently [44,57,58]. The addition of 5% reactive MgO increased the compressive strength by 7.73 (for T1M5) and 13.1% (for T2M5), showing a general trend of increasing (Figure 17c), which can be attributed to the formation of fine Mg(OH)_2_ crystals in the mortars when reacted with water [24,31]. In general, the geopolymer solidifies quickly and generates C-S-H and C-A-S-H gels, which cause considerable chemical, autogenous, and drying shrinkages at the early stage and adding MgO is beneficial to compensate for these issues. The generated large number of well-dispersed fine worm-like Mg(OH)_2_ crystals in the geopolymer’s high-alkalinity liquid-phase matrix environment do not overgrow, resulting in uniform volume/micro expansion, refining the pore size, effectively compensating for volume shrinkage in the hardening process, matching the shrinkage process, and enhancing the compressive strength, as observed in this study [24,31].

**Figure 18:**
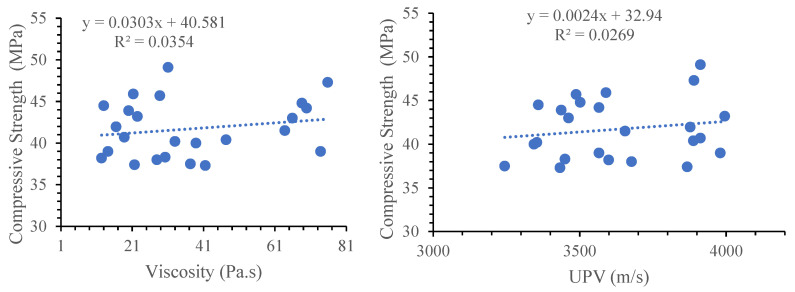
Correlation between compressive strength and viscosity or UPV.

The UPV is a function of the density with denser material (with a high viscosity and refined pore system) showing a high UPV and compressive strength. Despite a weak correlation, the general trend in Figure 18 shows an increase in the compressive strength with an increase in the viscosity and UPV.

The morphology and microstructural characteristics of the AAMs studied under a scanning electron microscope (SEM) during previous studies [22,61,62,63] showed a denser morphology for binary AAM mixes with a considerably smaller amount of un-hydrated/partially hydrated fly ash (FA) particles as compared to those of ternary mortars. This contributed towards the higher compressive strength for binary mixes. A high calcium content present in the formed geopolymer media contributes towards C-A-S-H formation, which, eventually, contributes to improving the strength [22,61]. In general, binary AAM mixes, due to their comparatively denser media and lesser un-hydrated FA, exhibited higher compressive strength values. Moreover, as pointed out in Table 3, the CaO/SiO_2_ ratio in all binary mixes is higher than the ternary ones. This signifies the presence of a high-calcium media to form AAM mixes with a considerably higher compressive strength. As for the comparison among mixes, reagent type 2 containing a higher amount of Ca(OH)_2_ is expected to contribute towards better strength development than reagent type 1. However, high silica and alumina content (based on the ratios shown in Table 3) from reagent type 1 can provide a substantial source for forming aluminosilicate, contributing towards geopolymer gel formation [22,61,62,63]. On the contrary, the ternary mix with reagent type 2 shows a better performance than its reagent type 1 counterpart. This can be considered due to the dissolution of higher silica and alumina due to the addition of low-calcium FA [22,61,62,63].

In comparison with the control mixes, AAM mixes with 5% MgO contain almost identical CaO/SiO_2_. However, the higher silica and alumina content corresponding to the mixes with MgO is a bit higher. Moreover, the addition of finer particles as MgO resulted in a particle with a higher surface area. This, eventually, leads to a higher water absorption on the particle surface, leaving a less porous and denser matrix to work with. These primary reasons contribute towards an increase in the compressive strength up to 43–44 MPa due to the introduction of MgO as an additive to the matrix. Moreover, the lesser porosity and dense matrix of these matrices can be further validated from a higher UPV and viscosity value (Table 3).

As pointed out by Li et al. [39], among three influencing factors, the substitution content of pozzolanic material has a higher impact as compared to the MWCNT content. This is due to the formation of N(Ca)-A-S-H and C-A-S-H polycondensation products, which contribute to the hydration and strength gaining at the later age [38,39]. Moreover, they contribute further by reducing the porosity and forming a denser matrix. For functionalized MWCNTs, the functionalized MWCNTs with carboxyl groups (-COOH) work as nucleation sites and supply many reaction sites for the formation of a higher amount of geopolymerization and hydration products. Therefore, the pore structure became denser, resulting in a higher UPV as well as viscosity values, as shown in Table 3. This denser matrix then yields a comparatively better compressive strength. However, Li et al. also suggested the usage of an MWCNT higher than 0.1% may result in a matrix with poorly dispersed MWCNT, which will lead to “reunification” of the matrix. These matrices, at later ages, become associated with many internal defects, leading to a significant strength reduction. During this study, the increase in the MWCNT content to 0.6% (%wt. by binder) did not show a significant improvement in the mechanical properties. Similarly, the incorporation of rGO increases the viscosity of the matrix due to agglomeration and due to the higher surface area of finer particles. Thus, the strength is expected to improve but only up to a certain extent. The increase in the rGO content here seemed to inversely affect the strength development. This can arguably be attributed to the lesser water affinity of the rGO group, leading to a higher localized water/binder ratio leading to a porous matrix.

## 4. Conclusions

This experimental study evaluates the effect of MgO, MWCNT, and rGO on the fresh state and rheological properties of developed ambient-cured one-part AAM mixes incorporating two types of high-calcium activators and the binary/ternary combination of precursors. The fresh state (in terms of the slump flow variation with time and setting times), rheological (in terms of viscosity and yield stress variations), and mechanical (compressive strength and UPV) properties were evaluated along with microstructural characteristics. The following conclusions are drawn from this study:

1. Both binary and ternary AAM mixes with 5% MgO showed about 30–35% higher viscosity and 2–2.25 times higher yield stress values compared to the controls (without MgO). This can be attributed to the higher surface area, water absorption, and formation of the hydrotalcite phase due to MgO addition.
2. AAM mixes incorporating MWCNT from 0% to 0.6% showed higher viscosity and yield stress values due to higher water affinity or/and the presence of an amorphous phase. Both the viscosity and yield stress values were increased for AAMs with 0.3% and 0.6% rGO, while the values were lower for 0.6% compared to 0.3%. A similar trend of variation in the viscosity and yield stress was noted for all the binary and ternary mixes with the binary mixes showing higher values compared to their ternary counterparts.
3. Variation in the viscosity and yield stress was insignificant for the AAM mixes with rGO; however, the values decreased with an increase in rGO from 0.3% to 0.6% due to the low water affinity of rGO.
4. The addition of MgO and MWCNT increased viscosity, decreased slump flow diameter and reduced setting time of AAMs, which can be attributed to an accelerated reaction resulting in a viscous media.
5. All the AAM mixes showed shear-thinning characteristics when subjected to a hysteresis test under varying shear strains. This can be attributed to the higher effects of the shearing stress produced from the hydrodynamic motion of the viscous-activating media than the re-flocculation of particles with a short-range interaction.
6. The 28-day compressive strength of the AAMs ranged from 37 MPa to 49 MPa. The addition of 5% MgO increased the compressive strength by 8 to 13%, while the use of WCNT/rGO showed an increasing trend up to 0.3% and a decrease at a higher dosage (0.6%).
7. Reasonable relations are found between the compressive strength, viscosity, and UPV, as well as the viscosity, slump flow, and setting time. The relation of the viscosity with the yield stress validates the fact that higher shear forces are required for highly viscous material required to induce inter-particle movements. Similarly, the variation in the viscosity against the slump flow showed inverse and good relations at 0 min, 30 min, and 60 min with R^2^ values of 0.812, 0.748, and 0.735, respectively.
8. Overall, the addition of MgO and MWCNT induced a higher viscosity associated with a low slump flow and high yield stress in AAMs, whereas the effect of the inclusion of rGO- was substantial. An MWCNT and rGO content of up to 0.3% provides nucleation sites, producing more hydration products, a denser matrix, higher compressive strength, and UPV.
9. The fresh state, rheological, and strength properties of AAMs are found to be influenced by the type and combinations of the source materials, reagents, and nanomaterials. The results will provide some guidelines for choosing combinations of these material parameters to design a mix for achieving the desired properties. A study comprising more variations in the MgO/rGO content and MgO-MWCNT-rGO combinations are recommended to obtain further holistic facts to improve the mix design and associated properties.

## Figures and Tables

**Table 1:** Chemical composition and physical characteristics of materials.

Chemical Composition (%)	Fly Ash Type C (FA-C)	Fly Ash Type F (FA-F)	Ground Granulated Blast-Furnace Slag (GGBFS)	Silica Sand	Magnesium Oxide (MgO)
SiO_2_	36.53	55.66	35.97	99.7	2.02
Al_2_O_3_	18.26	22.09	9.18	0.14	6.124
Fe_2_O_3_	5.66	4.26	0.50	0.016	0.94
CaO	20.97	7.97	38.61	0.01	2.4
MgO	5.08	1.16	10.99	0.01	92.26
K_2_O	0.68	1.49	0.36	0.04	-
Na_2_O	4.04	4.10	0.28	0.01	-
MnO	0.03	0.03	0.25	0	-
TiO_2_	1.26	0.61	0.39	0	-
P_2_O_5_	0.96	0.43	0.01	0	-
L.O.I.	2.18	1.05	0.74	0	1.14
Density (g/cm^3^)	2.61	2.02	2.87	2.65	3.58
Retained on 45 µ, %	-	18	-	3	
Blaine fineness (m^2^/kg)	315	306	489.30	-	

**Table 2:** Mix proportions for one-part alkali-activated mortars.

AAM Mix ID	Total SM	MgO/MWCNT /rGO	SM			Activator Type	Activator/Binder	Water/Binder
			FA-C	FA-F	GGBFS			
Control AAMs: without MgO, MWCNT, and rGO								
B1:B1M0/C0/R0	1	0	0.55	0	0.45	1	0.09	0.375
B2:B2M0/C0/R0	1	0	0.55	0	0.45	2	0.12	0.375
T1:T1M0/C0/R0	1	0	0.25	0.35	0.40	1	0.09	0.35
T2:T2M0/C0/R0	1	0	0.25	0.35	0.40	2	0.12	0.35
AAMs: with 5% MgO								
B1M5	1	0.05	0.52	0	0.43	1	0.09	0.375
B2M5	1	0.05	0.52	0	0.43	2	0.12	0.375
T1M5	1	0.05	0.24	0.33	0.38	1	0.09	0.35
T2M5	1	0.05	0.24	0.33	0.38	2	0.12	0.35
AAMs: with 0.3% and 0.6% MWCNT								
B1C3	1	0.03	0.55	0	0.45	1	0.09	0.375
B2C3	1	0.03	0.55	0	0.45	2	0.12	0.375
T1C3	1	0.03	0.25	0.35	0.40	1	0.09	0.35
T2C3	1	0.03	0.25	0.35	0.40	2	0.12	0.35
B1C6	1	0.06	0.55	0	0.45	1	0.09	0.375
B2C6	1	0.06	0.55	0	0.45	2	0.12	0.375
T1C6	1	0.06	0.25	0.35	0.40	1	0.09	0.35
T2C6	1	0.06	0.25	0.35	0.40	2	0.12	0.35
AAMs: with 0.3% and 0.6% rGO								
B1R3	1	0.03	0.55	0	0.45	1	0.09	0.375
B2R3	1	0.03	0.55	0	0.45	2	0.12	0.375
T1R3	1	0.03	0.25	0.35	0.40	1	0.09	0.35
T2R3	1	0.03	0.25	0.35	0.40	2	0.12	0.35
B1R6	1	0.06	0.55	0	0.45	1	0.09	0.375
B2R6	1	0.06	0.55	0	0.45	2	0.12	0.375
T1R6	1	0.06	0.25	0.35	0.40	1	0.09	0.35
T2R6	1	0.06	0.25	0.35	0.40	2	0.12	0.35

All numbers in mix design are mass ratios of binder; binder denotes source materials (SMs)/supplementary cementitious materials (SMs) such as FA-C, FA-F, GGBFS, and activator; MgO: magnesium oxide; MWCNT: multiwall carbon nanotube; rGO: reduced graphene oxide; B1: binary (FA-C + GGFS) AAM with activator type 1; B2: binary AAM with activator type 2; T1: ternary (FAC + FA-F + GGBFS) AAM with activator type 1; T2: ternary AAM with activator type 2; M5: AAM mixes with 5% MgO; C3/C6: AAM mixes with MWCNT of 0.3% and 0.6% wt. of binder; R3/R6: AAM mixes with rGO of 0.3% and 0.6% wt. of binder. Silica sand = 0.3 and HRWRA = 0.02 were used in the mix design.

**Table 3:** Chemical ratios, fresh state, rheological, and mechanical properties of AAM mixes.

AAM Mix ID	Chemical Ratios (SCMs + Reagent)				Mini-Slump Flow (mm) (0 s)	Viscosity (Pa.s) (0 s)	Yield Stress (Pa)	Setting Time (min)		Ultrasonic Pulse Velocity (UPV) * (m/s)	28-Day Compressive Strength (MPa) *
	SiO_2_/Al_2_O_3_	Na_2_O/SiO_2_	CaO/SiO_2_	Na_2_O/Al_2_O_3_				Initial	Final		
B1M0/C0/R0	2.62	0.09	0.84	0.23	200	65.91	160.8	216	248	3462	43.0
B2M0/C0/R0	2.56	0.14	1.02	0.35	250	13.03	62.2	311	369	3359	44.5
T1M0/C0/R0	2.75	0.08	0.59	0.22	245	37.31	77.4	145	230	3244	37.5
T2M0/C0/R0	2.69	0.12	0.73	0.32	300	12.42	54.4	458	483	3599	38.2
B1M5	2.58	0.09	0.85	0.23	180	75.81	267.2	170	240	3890	47.3
B2M5	2.51	0.14	1.03	0.35	215	31.05	182	215	240	3912	49.1
T1M5	2.97	0.05	0.54	0.14	235	47.3	224	200	270	3888	40.4
T2M5	2.97	0.05	0.54	0.14	285	22.47	98.7	410	450	3995	43.2
B1C3	2.62	0.09	0.84	0.23	200	69.89	185.7	200	240	3566	44.2
B2C3	2.56	0.14	1.02	0.35	220	28.76	88.4	300	350	3488	45.7
T1C3	2.75	0.08	0.59	0.22	230	38.87	91.3	110	195	3344	40.0
T2C3	2.69	0.12	0.73	0.32	290	18.8	62.4	330	405	3912	40.7
B1C6	2.62	0.09	0.84	0.23	175	73.8	207.4	190	230	3566	39.0
B2C6	2.56	0.14	1.02	0.35	210	27.9	169	285	335	3677	38.0
T1C6	2.75	0.08	0.59	0.22	215	41.5	103	95	170	3433	37.3
T2C6	2.69	0.12	0.73	0.32	270	21.67	87.8	327	402	3867	37.4
B1R3	2.62	0.09	0.84	0.23	190	68.57	215.6	210	245	3502	44.8
B2R3	2.56	0.14	1.02	0.35	215	21.3	107.3	305	355	3590	45.9
T1R3	2.75	0.08	0.59	0.22	225	32.98	113.8	110	200	3354	40.2
T2R3	2.69	0.12	0.73	0.32	275	16.5	72.3	335	410	3877	42.0
B1R6	2.62	0.09	0.84	0.23	190	63.8	189.7	200	235	3655	41.5
B2R6	2.56	0.14	1.02	0.35	220	20	97.2	290	400	3437	43.9
T1R6	2.75	0.08	0.59	0.22	210	30.25	110	105	200	3450	38.3
T2R6	2.69	0.12	0.73	0.32	280	14.2	67	330	410	3980	39.0

* mean values of three readings or samples; compressive strength (±1 MPa); UPV (±2 m/s).

## Data Availability

All data generated or analyzed during this study are included in this published article.
